# Minimally invasive micro sclerostomy (MIMS) procedure in the treatment of open-angle glaucoma

**DOI:** 10.1186/s12886-024-03384-y

**Published:** 2024-03-18

**Authors:** Lilit Voskanyan, Iqbal Ike K. Ahmed, Assaf Gershoni, Edward Barayev, Vahan Papoyan, Astghik Ghazaryan, Oren Bar-Ilan, Alon Zahavi, Yoseph Glovinsky, Noa Geffen

**Affiliations:** 1 Ophthalmological Center after S.V. Malayan, Yerevan, Armenia; 2 Mikhitar Heratsi Yerevan State Medical University, Yerevan, Armenia; 3 Department of Ophthalmology and Vision Sciences, University of Toronto, Toronto, ON Canada; 4 Department of Ophthalmology, Rabin Medical Center– Beilinson Hospital, 39 Jabotinski St, Petach Tikva, Israel; 5 Ophthalmology Department and Laboratory of Eye Research, Felsenstein Medical Research Center, Rabin Medical Center, Petach Tikva, Israel; 6 Faculty of Medicine, Tel Aviv University, Tel Aviv, Israel; 7 DataSights Ltd, Enfield, England, UK; 8 Goldschleger Eye Institute, Sheba Medical Center, Tel Hashomer, Israel

**Keywords:** Glaucoma, MIGS, Minimally invasive micro sclerostomy, MIMS

## Abstract

**Background:**

To evaluate the safety and efficacy of the Minimally Invasive Micro Sclerotomy (MIMS) procedure in the management of uncontrolled open-angle glaucoma.

**Methods:**

A prospective, open-label, single-arm clinical evaluation with intra-subject comparisons performed at the Ophthalmologic Center after S.V. Malayan, Yerevan, Armenia. Included were adults with primary open-angle glaucoma (OAG) (*N* = 114) or exfoliative glaucoma (*N* = 6) who were uncontrolled (IOP > 21) on tolerated topical medication. Mild (*N* = 7), moderate (*N* = 66) and severe (*n* = 47) cases were prospectively included without preselection. Following subconjunctival Mitomycin C, an ab-interno MIMS procedure was performed alone (*N* = 100) or combined with phacoemulsification (*N* = 20). Patients were followed for 52 weeks. Procedure-related complications and adverse events were recorded. Success criteria were defined as -5 < IOP ≤ 21mmHg OR a reduction in IOP of ≥ 20% from baseline with (qualified success) or without (complete success) hypotensive medications.

**Results:**

Mean patient age was 69 ± 10.1 years. The mean duration of the procedure was 2:01 ± 0:41 min:sec. Scleral drainage channels were achieved in all cases. No device malfunctions, intraoperative complications, or serious adverse events were reported. Iris plugging of the sclerostomy site and early spikes in IOP were the most common adverse events. The only reason for failure was final IOP > 21 mmHg on tolerated medication. At 52 weeks (*n* = 93), mean IOP decreased by 38% from baseline (*P* < 0.001), from 27.9 ± 3.7 to 17.5 ± 5.3 mmHg, a difference of 10.5 mmHg (95% CI: -11.7, -9.3). One-year qualified success was documented in 82.1% (95% CI: 72.9%,89.2%) of the patients and complete success, in 70.5% (60.3-79.4%). 60% (95 CI:49.4%,69.9%) of the patients achieved maximum IOP level of 14 mmHg or at least 30% reduction in IOP.

**Conclusions:**

MIMS procedure is a relatively simple, short and safe minimally invasive bleb-forming procedure. Its efficacy, as found in this short-term evaluation, lends it suitable for mild and moderate uncontrolled open-angle glaucoma patients.

**Trial registration:**

ClinicalTrials.gov ID: NCT04503590 2019-05-29.

## Introduction

Glaucoma is an optic neuropathy characterized by progressive degeneration of the retinal ganglion cells [1]. It is the leading cause of irreversible blindness worldwide [2]. The number of people with open-angle glaucoma (OAG) and angle-closure glaucoma rose from 60.5 million in 2010 to 79.6 million in 2020 and is projected to increase to 111.8 million by 2040 [3, 4]. Although glaucoma is a multifactorial disease, reducing the associated elevated intraocular pressure (IOP) is the only means to restrain its progress. Initial glaucoma treatment is generally based on topical eye drops and laser trabeculoplasty. Incisional surgeries, such as trabeculectomy and glaucoma drainage device implantation, have been shown to effectively lower IOP [5–8], but they have several important disadvantages. Besides their long learning curve, both surgical and recovery times are long, frequent follow-up is necessary, and they result in labile postoperative IOPs and other adverse events [9]. Therefore, these procedures are often reserved for patients with more severe disease who warrant aggressive intervention.

In the last decade, minimally invasive glaucoma surgery (MIGS) has been gaining popularity. MIGS encompasses a variety of microsurgeries that can be categorized by their mechanism of action: enhancing flow through the trabecular meshwork, shunting aqueous humor to the subconjunctival or suprachoroidal space, and decreasing aqueous humor production. Good results with minimal trauma have been reported, in addition to a high safety profile, relatively short operating time, and quick patient recovery [10, 11]. In most cases, MIGS can be combined with cataract surgery. However, as the reduction in IOP is generally of lesser magnitude than with trabeculectomy or even glaucoma drainage devices (GDDs), MIGS is generally considered suitable only for patients with mild to moderate disease in whom IOP remains uncontrolled despite medical and laser therapy, and for patients with poor adherence, intolerance, or limited access to medical treatment with otherwise limited options [12, 13].

Minimally Invasive Micro Sclerostomy (MIMS), developed by Sanoculis Ltd. (Kiryat Ono, Israel), is a novel ab interno stent-less MIGS procedure. It creates a drainage channel with a diameter of 100 microns extending from the anterior chamber to the subconjunctival space. The preclinical trials performed in vitro and in an in vivo experimental porcine model consistently yielded relatively high safety, feasibility, and efficacy profiles [14]. These were followed by a clinical, prospective, open-label, single-arm trial that included 31 patients who underwent either a stand-alone MIMS procedure (*n* = 10) or a combined phacoemulsification-MIMS procedure (*n* = 21). As reported by Geffen et al. [15] the interim results suggested that the MIMS procedure may serve as a simple, safe, and effective surgical alternative for patients with early OAG with a target IOP in the mid-to-high teens. The purpose of the present study was to implement the conclusions drawn from the first clinical trial and to further investigate the safety, performance, and efficacy of MIMS in a larger group of patients.

## Methods

### Setting and design

A prospective, single-center, open-label, single-arm, nonrandomized clinical evaluation with intra-subject comparisons was conducted in accordance with CONSORT guidelines at the Ophthalmological Center after S.V. Malayan, Yerevan, Armenia. After evaluating the safety, necessity and quality of the study protocol, it was approved by the local ethics committee (Ophthalmological Center after S.V. Malayan, MMS EEU-1, SN 01203444). All procedures were performed in accordance with the standards of the responsible committee on human experimentation (institutional and national) and the Helsinki Declaration of 1975, as revised in 2000 and 2008. Informed consent to participate in the study was obtained from all patients. Participation was voluntary, and patients were allowed to withdraw from the study at any time. The clinical trial registration number was NCT04503590 (Clinicaltrials.gov). Patients were enrolled and operated between May 2019 and February 2020.

### Patient selection

The cohort consisted of patients with uncontrolled OAG, including primary OAG or pseudoexfoliative glaucoma, in which IOP could not be reduced below 22mmHg by tolerated topical medical treatment. Patients were referred for the MIMS procedure only when, in the opinion of the investigator, lowering their IOP to the range between 6 and 21 was needed to control their disease. Glaucoma was diagnosed by a senior glaucoma specialist based on the structural and functional characteristics described by Foster et al. [16].

Inclusion criteria were age at least 18 years, preoperative best corrected visual acuity (BCVA) ≥ 20/30 (Snellen equivalent), and ability and willingness to provide informed consent and attend follow-up visits through 1 year postoperatively. In patients with two eligible eyes, only the first eye undergoing surgical treatment was included in the analysis. MIMS was performed with or without cataract surgery. Patients underwent stand-alone MIMS if cataract was not present or was considered nonsignificant in the study eye and Shaffer grade ≥ III was observed in all four angle quadrants. Patients with an uncontrolled glaucoma who had also vision disturbing cataract in the study eye, underwent combined MIMS and phacoemulsification with intraocular lens (IOL) implantation.

Exclusion criteria were glaucoma other than primary OAG, pigmentary glaucoma, and pseudoexfoliative glaucoma, angle abnormalities, ocular pathology that could interfere with accurate IOP measurements, history of significant ocular trauma, any significant ocular comorbid disease, previous surgery in the study eye except for clear corneal cataract extraction with IOL implantation within the capsular bag that was performed > 6 months prior to recruitment, laser trabeculoplasty within 90 days before the screening visit, presence of peripheral anterior synechiae, active corneal disease (inflammation, infection or edema) or corneal opacities/disorders inhibiting visualization of the angle, elevated episcleral venous pressure, history of uveitis or infection within 90 days before screening in either eye, and BCVA below 20/50 (Snellen equivalent) in the fellow eye or a clinically significant ocular pathology in the fellow eye. Also excluded were patients who were using oral hypotensive medication for glaucoma treatment in the fellow eye, had uncontrolled systemic disease that, in the opinion of the investigator, would put the subject’s health at risk and/or prevent the subject from completing all study visits, were pregnant or lactating, who had participated in another clinical trial within 90 days before screening. Although discontinuation of blood thinners is usually left for surgeon-discretion, it was decided not to include patients with a higher risk for bleeding, who could not discontinue blood thinners use, to achieve standardized conditions.

For subjects in whom combined phacoemulsification-MIMS surgery was indicated, additional exclusion criteria were BCVA < 20/40 (Snellen equivalent) in the fellow eye, corneal opacity that could interfere with cataract surgery, extremely dense cataract, traumatic cataract, bag instability, and increased risk of corneal decompensation, such as Fuchs endothelial corneal dystrophy with endothelial cell count less than 1000 mm^2^ or central pachymetry > 600 microns. Intraoperatively, MIMS was not performed if the cataract procedure was complicated by a conjunctival tear or Descemet membrane detachment that could interfere with the MIMS procedure and/or posterior capsular rupture with vitreous loss and/or a dropped nucleus or lens fragment, and/or need to position the IOL anywhere else but within the capsular bag, for example, in the sulcus or anterior chamber or fixated to the sclera or iris.

### MIMS procedure

Ab interno MIMS was performed under topical (1% tetracaine) and subconjunctival (2% lidocaine) anesthesia, whether as a stand-alone procedure or in combination with clear corneal incision cataract surgery using phacoemulsification and posterior-chamber IOL implantation. All patients were operated with the MIMS system prototype no: MMS1000 MIMS DEVICE (Sanoculis Ltd., Israel). The surgical systems include a disposable hand piece and the MIMS activation device. The full surgical technique was performed as described by Geffen at al [15].. In all cases, a subconjunctival injection of 0.1 mL Mitomycin C (MMC) 0.02% was given 1 h before the procedure. Ocular viscoelastic agent (Viscoat, Alcon Laboratories, Inc., Fort Worth, TX) was injected into the subconjunctival space at the superonasal quadrant for potential accommodation of the protruded surgical tool during the procedure. A temporal paracentesis of 1.5 mm was created. Following injection of approximately 0.2 mL of ocular viscoelastic agent (Viscoat) into the anterior chamber, the MIMS surgical tool was introduced into the anterior chamber. The tip was positioned at the superior angle, above the trabecular meshwork, and the system was operated. A thin cylinder of sclero-corneal tissue was removed, creating a drainage channel connecting the anterior chamber to the subconjunctival space. At the end of the procedure, partial washout of the viscoelastic agent was performed,, and finally, ciprofloxacin 0.3% and dexamethasone 0.1% drops were instilled.

Postoperatively, patients were prescribed topical ciprofloxacin 0.3% and dexamethasone 0.1% drops for 4 weeks with gradual tapering-down. The pupil was constricted with pilocarpine 2% eyedrops administered for 2 weeks following the procedure to minimize the risk of iris plugging of the internal ostium of the channel. Glaucoma medications (Cosopt or Azarga) were added as per protocol, whenever IOP record was above 21 mmHg. Trabeculectomy was performed if IOP remained uncontrolled for more than a month despite addition of medications.

### Study protocol

Patients were examined at baseline (screening visit) and 1 day, 1 week (± 2 day), 2 weeks (± 3 days), 4 weeks (± 7 days), 12 weeks (± 14 days), 24 weeks (± 14 days), 36 weeks (± 14 days), and 52 weeks (± 21 days) after surgery. All additional unscheduled visits were recorded and reported as such within the Case Report Forms. Measurements performed at baseline included automated refraction test, BCVA evaluation using an ETDRS chart, a comprehensive biomicroscopic examination including IOP, gonioscopy, a dilated fundus examination with optic disc assessment, and a thorough retinal examination directed toward identifying pathologies that could exclude the eye from the study.

All IOP measurements were performed using a calibrated Goldmann applanation tonometer (Haag Streit, Berne, Switzerland) during the morning hours (08:00–10:00 am). Two IOP measurements were taken at each study visit and the average was recorded; a third IOP measurement was taken if the difference between the first two was more than 3 mmHg and the average of the two closest measurements was recorded. Central corneal thickness was recorded with optical coherence tomography (OCT) (Optovue, iVue 100-2, Haag Streit, Koeniz, Switzerland).

On the day of surgery, the investigators recorded the duration of the procedure, the performance of the MIMS system, and all intraoperative complications.

Examinations performed during the postoperative visits included BCVA testing, Goldmann applanation tonometry and biomicroscopy. Seidel’s test was performed, and morphologic bleb features were documented, as well as the ocular medications being used. At baseline, and starting at the 2-week postoperative visit, patients underwent gonioscopy, specular microscopy (Konan specular microscope XVII, Cellchek 20, Konan Medical, Irvina, CA, USA), and anterior segment and macular OCT (Optovue, model iVue 100-2, Haag Streit, Koeniz, Switzerland). The OCT was also used to evaluate the bleb configuration including height and extent.

### Outcome measures

System performance was assessed by the integrity of the drainage channel created at the sclera-corneal junction, extending from the anterior chamber to the subconjunctival space, as indicated on anterior segment OCT. Assessment of the safety of the procedure was based on the records of complications and adverse events that occurred during surgery and thereafter, documented by the investigators. Assessment of efficacy was based on the proportion of patients meeting the criteria of three different definitions of complete and qualified success. The primary outcome measure was the rate of complete success at 52 weeks, defined as 5 < IOP ≤ 21 mmHg OR IOP reduction of > 20% from baseline AND no need for filtration surgery or hypotensive medication. Qualified success was defined as 5 < IOP ≤ 21 mmHg OR IOP reduction of > 20% from baseline AND no need for filtration surgery, with the same or smaller number of hypotensive medications.

The secondary outcome measure was the rate of complete success defined as 5 < IOP ≤ 18 mmHg, OR IOP reduction of > 25% from baseline AND no need for filtration surgery or hypotensive medication. Qualified success was defined as 5 < IOP ≤ 18 mmHg OR IOP reduction of > 25% from baseline AND no need for filtration surgery, with the same or smaller number of hypotensive medications.

The tertiary outcome measure was the rate of complete success defined as 5 < IOP ≤ 14 mmHg OR IOP reduction of > 30% from baseline AND no need for filtration surgery or hypotensive medication, Qualified success was defined as 5 < IOP ≤ 14 mmHg OR IOP reduction of > 30% compared to baseline AND no need for filtration surgery, with the same or a smaller number of hypotensive medications.

Failure was defined by any of the following criteria: final IOP outside the aforementioned range, development of any serious complication, severe loss of vision, or need to undergo additional glaucoma surgery other than bleb needling or laser iridoplasty to retract the iris from the internal ostium of the channel.

### Statistical analysis

For data analysis, continuous variables were described by means and standard deviations (SD). Dichotomous variables were presented as percentages with 95% confidence interval. Paired t-test was used to compare IOP measurements after testing the distribution of IOP differences for normality (Jarque-Bera test). The reduction in the number of medications between baseline and the final visit was tested for significance by the Wilcoxon signed rank test for paired observations. This test was also used for paired comparison of IOP in the Phacoemulsification-MIMS subgroup which had less than 30 patients. All statistical analyses were performed using JMP® Pro Statistical Discovery software, version 15.2.1 (SAS Institute Inc., Cary, NC, USA). *P*-values < 0.05 were considered statistically significant.

## Results

The cohort consisted of 120 patients (120 eyes): 114 diagnosed with uncontrolled primary OAG and 6 with pseudoexfoliative glaucoma. Glaucoma severity as determined by the cup to disc (CD) ratio was mild (CD ratio 0.5–0.6) in 7 patients, moderate in 66 patients (CD ratio 0.7–0.8) and severe (0.9-1.0) in 47 patients. There were 70 men and 50 women of mean age 69.1 ± 10 years. Stand-alone MIMS was performed in 100 patients, and combined MIMS with phacoemulsification and IOL implantation, in 20.In 20/120 cases cataract severity demanded a combined phacoemulsification-MIMS procedure while all others underwent a stand-alone MIMS procedure. All operations were performed by two surgeons (L.V. [*N* = 110] and I.I.K.A. [*N* = 10]). The left eye was operated in 65 patients (54%), and the right, in the remainder. Mean duration of the MIMS procedure was 2:01 ± 0:41 min:sec (range 0:52 to 5:00 min). No device malfunctions were recorded.

Data of patients who did not attend either the 24- or 52-week follow-up visit were excluded from the performance analysis. Data of all patients were included in the safety analysis. Of the total 120 patients, 93 (77.5%) completed 52 weeks of follow-up.

### Efficacy

#### Drainage-channel creation and filtering-bleb formation

**Fig. 1:**
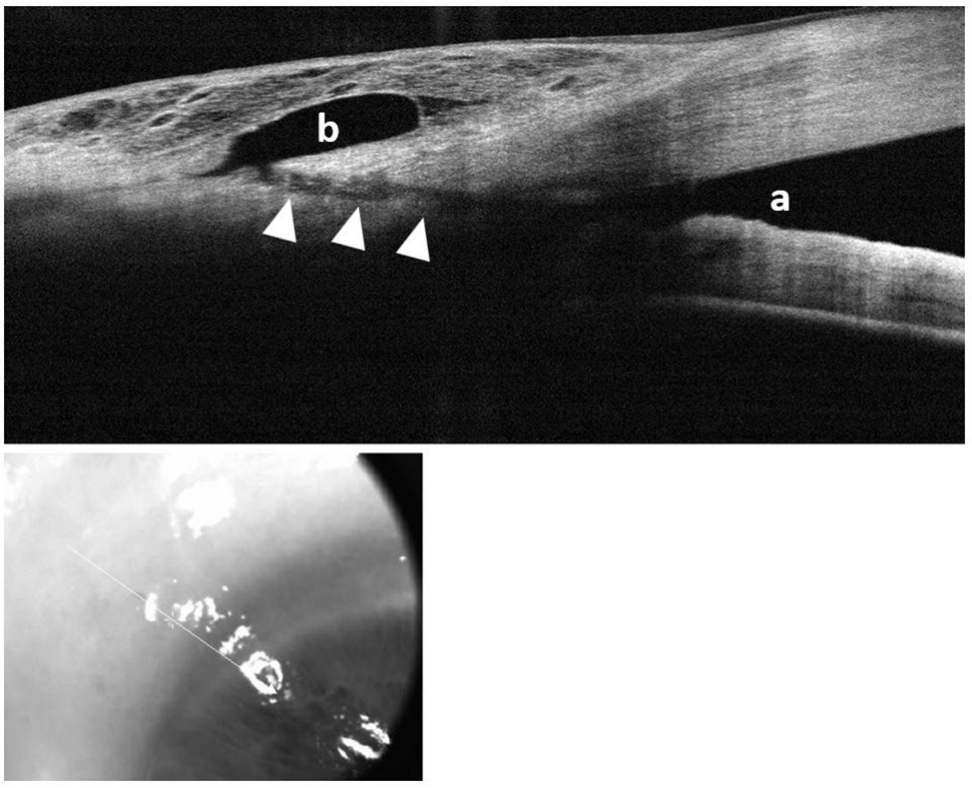
Anterior-segment OCT scan (top) with corresponding grayscale photograph produced from the Optovue OCT device, 52 weeks following stand-alone MIMS procedure. Drainage from the anterior chamber angle (**a**) is achieved through the scleral tunnel (arrowheads) to a subconjunctival filtering bleb (**b**). As shown in the photograph below, the bleb itself is relatively shallow and mildly vascularized

Anterior segment OCT examination showed that a scleral tunnel was successfully created with the MIMS system in all cases. Mean tunnel diameter was 109 ± 17 μm (range 81–140), and mean tunnel length, 1288 ± 309 μm (range 905–2181). Anterior segment OCT imaging proved bleb formation, indicating that most blebs were diffuse and relatively shallow, with mild vascularity (Fig. 1).

#### IOP and medication reduction

**Fig. 2:**
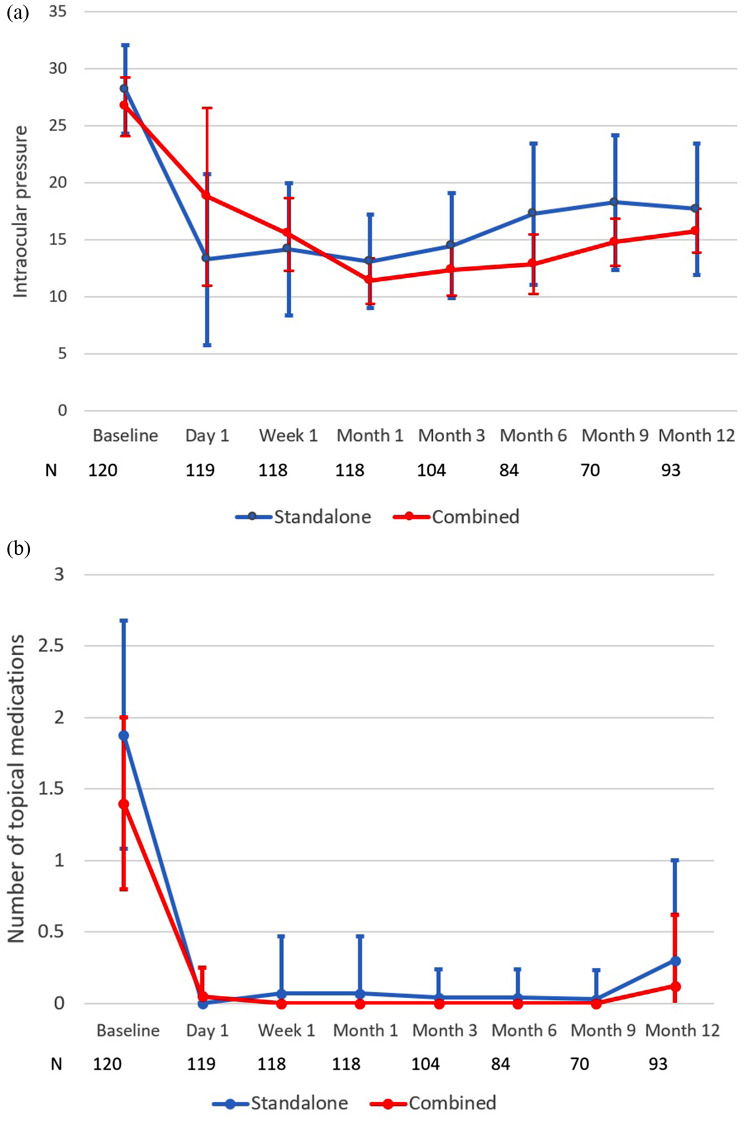
**a** Mean ± SD (mmHg) intraocular pressure of study participants at all study visits, in stand-alone MIMS patients and MIMS combined with phacoemulsification and IOL implantation. **b** Mean ± SD number of topical hypotensive medications of study participants at all study visits, in stand-alone MIMS patients and MIMS combined with phacoemulsification and IOL implantation

Table 1 presents the mean (± SD) IOP measurements and the mean (± SD) number of hypotensive medications being used at all consecutive study visits, for the whole cohort and by type of procedure (stand-alone MIMS or combined phacoemulsification-MIMS). Mean IOP at baseline (*n* = 120) was 27.9 ± 3.7 mmHg, dropping to 14.2 ± 7.8 mmHg on postoperative day 1 (*n* = 119), 14.4 ± 5.4 mmHg at 1 week (*n* = 118), 12.8 ± 3.9 mmHg at 4 weeks (*n* = 118), 14.1 ± 4.4 mmHg at 12 weeks (*n* = 104), 16.5 ± 6.0 mmHg at 24 weeks (*n* = 84), 17.6 ± 5.5 mmHg at 36 weeks (*n* = 70), and 17.3 ± 5.3 mmHg at 52 weeks (*n* = 93). At baseline, patients were using 1.8 ± 0.8 hypotensive medications, dropping to 0.01 ± 0.1 on postoperative day 1, 0.06 ± 0.3 at 1 week, 0.06 ± 0.3 at 4 weeks, 0.04 ± 0.2 at 12 weeks, 0.05 ± 0.2 at 24 weeks, 0.04 ± 0.2 at 36 weeks, and 0.27 ± 0.7 at 52 weeks (Fig. 2). Statistical analysis upon completion of the study showed that the difference from baseline for IOP and medication was statistically significant (*P* < 0.001 for all).

**Table 1:** Mean IOP and mean number of hypotensive medications during follow-up in patients treated with MIMS

All patients	Study visit							
	Baseline	Day 1	Week 1	Week 4	Week 12	Week 24	Week 36	Week 52
N	120	119	118	118	105	86	72	95
IOP (mmHg), mean ± SD	27.9 ± 3.7	14.2 ± 7.8	14.4 ± 5.4	12.8 ± 3.9	14.1 ± 4.4	16.5 ± 6.0	17.6 ± 5.5	17.3 ± 5.3
P value (vs. baseline)	–	< 0.001	< 0.001	< 0.001	< 0.001	< 0.001	< 0.001	< 0.001
No. of medications, mean ± SD	1.80 ± 0.8	0.01 ± 0.1	0.06 ± 0.3	0.06 ± 0.3	0.04 ± 0.2	0.05 ± 0.2	0.04 ± 0.2	0.27 ± 0.7
P value (vs. baseline)	–	< 0.001	< 0.001	< 0.001	< 0.001	< 0.001	< 0.001	< 0.001
**Stand-alone MIMS**								
N	100	99	98	99	87	71	58	78
IOP (mmHg), mean ± SD	28.2 ± 3.9	13.3 ± 7.5	14.2 ± 5.8	13.1 ± 4.1	14.5 ± 4.6	17.3 ± 6.2	18.3 ± 5.9	17.7 ± 5.8
P value (vs. baseline)	–	< 0.001	< 0.001	< 0.001	< 0.001	< 0.001	< 0.001	< 0.001
No. of medications, mean ± SD	1.88 ± 0.8	0.00 ± 0.0	0.07 ± 0.4	0.07 ± 0.4	0.04 ± 0.2	0.04 ± 0.2	0.03 ± 0.2	0.30 ± 0.7
P value (vs. baseline)	–	< 0.001	< 0.001	< 0.001	< 0.001	< 0.001	< 0.001	< 0.001
**Phacoemulsification-MIMS**								
N	20	20	20	19	18	15	14	17
IOP (mmHg), mean ± SD	26.7 ± 2.6	18.8 ± 7.8	15.5 ± 3.2	11.4 ± 2.0	12.4 ± 2.3	12.9 ± 2.6	14.8 ± 2.1	15.8 ± 1.9
P value (vs. baseline)	–	–	–	–	–	–	–	< 0.001
No. of medications, mean ± SD	1.40 ± 0.6	0.05 ± 0.2	0.00 ± 0.0	0.00 ± 0.0	0.00 ± 0.0	0.00 ± 0.0	0.00 ± 0.0	0.12 ± 0.5
P value (vs. baseline)	–	–	–	–	–	–	–	< 0.001

IOP = intraocular pressure, MIMS = Minimally Invasive Micro Sclerostomy

#### Success and failure

Table 2 presents the cumulative success rates and 95 confidence intervals, as defined by three sets of criteria, in the whole cohort. Table 3 presents the same for the stand-alone subgroup.

**Table 2:** Success criteria at multiple IOP levels at study endpoints– whole study group

Study visit					
IOP levels (mmHg)	Success criteria	6 months		12 months	
		n/N (%)	95% CI	n/N (%)	95% CI
5 < IOP ≤ 21 OR ≥ 20% IOP reduction	Complete	68/86 (79)	70.9–88.7%	67/95 (71)	60.3–79.4%
	Qualified	72/86 (83)	76.4–92.4%	78/95 (82)	72.9–89.2%
5 < IOP ≤ 18 OR ≥ 25% IOP reduction	Complete	63/86 (73)	62.6–82.2%	63/95 (66)	55.9–75.7%
	Qualified	67/86 (78)	67.7–86.1%	73/95 (77)	67.1–84.9%
5 < IOP ≤ 14 OR ≥ 30% IOP reduction	Complete	58/86 (67)	56.5–77.2%	57/95 (60)	49.4–69.9%
	Qualified	61/86 (71)	60.1–80.2%	65/95 (68)	58.1–77.6%

IOP = Intraocular pressure. Complete success - % of patients with IOP in indicated range, or an IOP reduction in range, compared to baseline, with no need of filtration surgery or hypotensive medication. Qualified success - % of patients with IOP in range, or an IOP reduction in range, compared to baseline, and no need of filtration surgery, with or without hypotensive medication

**Table 3:** Success criteria at multiple IOP levels at study endpoints– standalone procedure

Study Visit					
IOP Levels (mmHg)	Success Criteria	6 months		12 months	
		n/N (%)	95% CI	n/N (%)	95% CI
5 < IOP ≤ 21 OR ≥ 20% IOP reduction	Complete	53/71 (75)	62.9–84.2%	50/78 (64)	52.4–74.7%
	Qualified	57/71 (80)	69.1–88.8%	62/78 (80)	68.8–87.8%
5 < IOP ≤ 18 OR ≥ 25% IOP reduction	Complete	48/71 (68)	55.5–78.2%	47/78 (60)	48.5–71.2%
	Qualified	52/71 (73)	61.4–83.1%	56/78 (72)	60.5–81.4%
5 < IOP ≤ 14 OR ≥ 30% IOP reduction	Complete	43/71 (61)	48.3–72%	41/78 (63)	40.9–64%
	Qualified	46/71 (65)	52.5–75.8%	48/78 (62)	49.8–72.3%

IOP = Intraocular pressure. Complete success - % of patients with IOP in indicated range, or an IOP reduction in range, compared to baseline, with no need of filtration surgery or hypotensive medication. Qualified success - % of patients with IOP in range, or an IOP reduction in range, compared to baseline, and no need of filtration surgery, with or without hypotensive medication

**Fig. 3:**
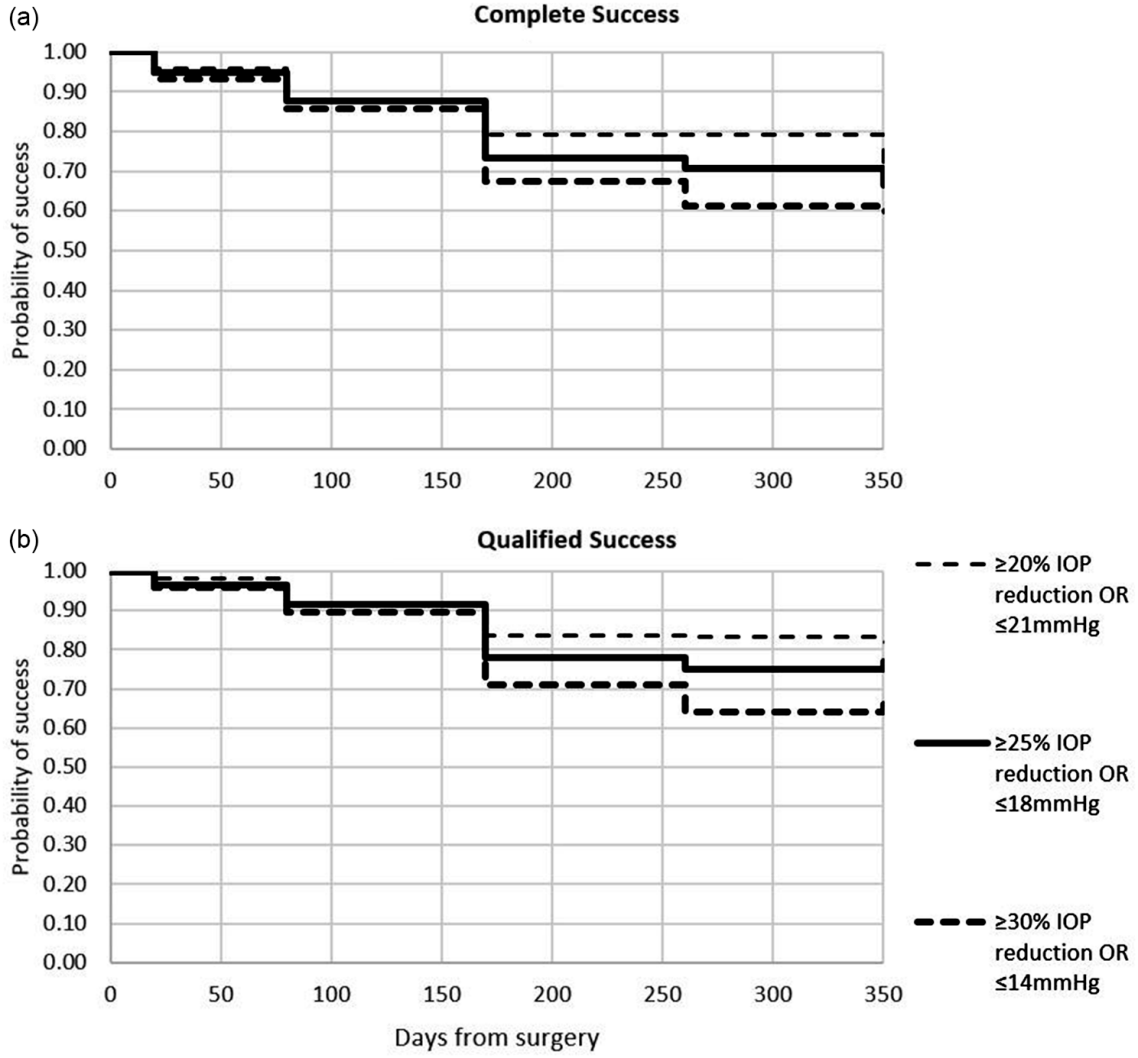
. Survival curves for complete (top) and qualified (bottom) success for primary (>20% IOP reduction or <21mmHg), secondary (>25 % IOP reduction or <18mmHg) and tertiary (>30 % IOP reduction or <14mmHg) success criteria

In the total study population, there were 24 failures (24.2%), all in the stand-alone MIMS group: 2 patients required a trabeculectomy procedure, and 20 had an IOP of > 21 mmHg at 12 months, including 5 who used hypertensive medications and 16 who did not. An additional case was considered a failure because although IOP was 14 mmHg at 12 months, the patient was using more hypotensive medications at 12 months than at baseline (1 and 2 medications, respectively). Survival curves for complete and qualified success are shown in Fig. 3.

### Safety

No serious ocular or systemic adverse events were reported during the study period. All adverse events were considered as mild to moderate. The most common was iris plugging of the internal ostium of the channel which occurred in 18 patients. In 7 cases it occurred early (≤ 12 weeks postoperatively) and in 11 late (> 12 weeks postoperatively). Treatment with topical pilocarpine drops followed by laser application on the adherent iris synechia led to complete resolution in all but 3 patients in whom persistent iris plugging was observed at the 52-week visit.

There were 15 cases with an IOP spike (> 30mmHg) on postoperative day one. This was attributed to retained viscoelastic material in the anterior chamber. All resolved at the slit-lamp by partial removal of the viscoelastic material by a gentle pressure on the posterior lip of the temporal paracentesis. All the above cases ended-up without sequelae, with or without topical treatment. One patient had 20% endothelial cell loss without corneal edema after a combined phacoemulsification-MIMS procedure which was attributed to the high nuclear density of the cataract, requiring high mean intraoperative cumulative dissipated energy during phacoemulsification. Four patients had early corneal edema which spontaneously resolved, and there were single events of mild hyphema, choroidal effusion, ciliary body detachment, and branch retinal vein occlusion. Overall, BCVA decreased over the study period by ≥ 2 lines in 11 patients (9.2%), all due to cataract progression, with no cases of irreversible vision loss.

Post-operative interventions: two patients underwent trabeculectomy procedure due to bleb scarring and elevated IOP (both considered as failure). One patient underwent a successful needling procedure with MMC.

## Discussion

The present one-year clinical evaluation provides initial information on the performance, safety, and efficacy of the MIMS procedure in the treatment of patients with OAG. Based on the lessons learned from the first human evaluation of MIMS in early 2022 [15], the surgical technique and postoperative management were improved. The present real life, case series evaluation was conducted in a larger cohort followed for 1 year. The procedure proved to be easy to learn and relatively quick to perform. No device malfunctions were recorded.

At 52 weeks’ follow-up, we found a mean reduction in IOP of 38.0% in MIMS-treated eyes (37.4% for MIMS stand-alone, and 40.6% for phacoemulsification-MIMS), and a mean reduction in number of glaucoma medications of 85.1% (83.9% for MIMS stand-alone, and 91.6% for MIMS-phacoemulsification). These values are comparable to those reported in the studies included in the 2017 systematic review and meta-analysis of Lavia et al. [17] investigating the effect of surgery with different MIGS devices on IOP and need for glaucoma medications at 1 year.

To evaluate the success of the procedure, we used three sets of criteria. Applying the first (primary outcome measure), we found that 70.5% (95% CI: 60.3%, 79.4%) of the patients achieved complete success (5 < IOP ≤ 21 mmHg OR IOP reduction of > 20% from baseline AND no need for filtration surgery or hypotensive medication) and 82.1%(95% CI: 72.9%, 89.2%) achieved a qualified success (criteria as above, however with the same or a smaller number of hypotensive medications). Using similar IOP definitions to assess the effectiveness of the PRESERFLO MicroShunt in 81 patients with primary OAG, Beckers et al. [18] reported a reduction in IOP from 21.7 ± 3.4 mmHg at baseline to 14.5 ± 4.6 mmHg at 1 year (*P* < 0.0001). The overall success rate (with and without supplemental glaucoma medication) was 74.1%, similar to our study. However, the PRESERFLO procedure entails substantial conjunctival dissection and stent implantation whereas the MIMS is stent-less and requires no conjunctival dissection and a much shorter surgical time.

The XEN Glaucoma Treatment System (Allergan, Abbvie Company, Irvine, CA, USA) is, like MIMS, a sub conjunctival filtration procedure [19]. In a study of XEN safety and efficacy, Wagner et al. [20] reported 1-year complete and qualified success rates of 58.5% and 70.7%, respectively, comparable to our results with MIMS. Others reported lower rates of 31% and 33%, respectively, albeit in association with mean IOP reductions of 23% and 45.8% [21], in line with rates in the present results. Mansouri et al. [19] prospectively compared the 1-year safety and efficacy of stand-alone XEN gel implantation with combined phacoemulsification-XEN in 149 eyes with OAG. Mean IOP under medication was 20.0 ± 7.1 mmHg at baseline and 13.9 ± 4.3 mmHg at 1 year (*P* < 0.01), for a 31% reduction, similar to our study, and a corresponding reduction in mean number of hypotensive medications from 1.9 ± 1.3 at baseline to 0.5 ± 0.8. In the present study, the mean number of hypotensive medications was 1.8 ± 0.8, and it was reduced to 0.27 ± 0.7 at 1 year after MIMS (*P* < 0.001). In total, 62.1% of patients achieved a ≥ 20% IOP reduction with the XEN implant, which is somewhat lower than with the MIMS procedure. The proportion for both XEN and MIMS procedures was higher in the stand-alone than the combined-procedure groups.

The mean IOP reduction reported for trabeculectomy in the literature ranges from 44 to 55% [22–26], greater than the 38% found here, although the trabeculectomy-associated reduction in glaucoma medications, ranging from 72 to 96% [20, 22–25], is comparable to our rates of 83.9% and 91.6% for MIMS stand-alone and phacoemulsification-MIMS, respectively. The complete success rate of 70.5% for MIMS after 52 weeks’ follow-up was also similar to the trabeculectomy rate of 57.9–84.8% [22, 24, 25].

Failure was documented in 23 patients (24%). Most were categorized as failure because their IOP was > 21 mmHg at 52 weeks. However, 16 of them (69%) did not use any hypotensive medications at the last visit compared to 2.1 medications prior to surgery. Their average IOP was 29 mmHg before surgery and 25.7 mmHg after. There is good reason to believe that had they used even one hypotensive medication, a substantial percentage of this subgroup might have been categorized as a qualified success.

The safety profile of glaucoma filtration procedures is relatively low. Iris plugging of the internal ostium of the channel was the most common adverse event in our study, occurring in both the early and late postoperative periods. Several corrective measures are available to avoid or minimize this complication, including leaving viscoelastic material to 50% fill in the anterior chamber to prevent early hypotony [27–29], preventing pressure from being applied on the eye, and using pilocarpine 2% eye drops for 2 weeks following the procedure to constrict the pupil and pull the iris away from the internal ostium [9, 30]. Close postoperative monitoring is recommended to ensure a timely reaction to plugging, if it occurs. Further research to determine the optimal sizing of the channel may be required.

Traditional glaucoma surgery, such as trabeculectomy or a GDD, carries a high risk of complications, some of which can be sight-threatening. Gedde et al. [23] reported the 1-year outcomes of 350 mm^2^ Baerveldt glaucoma implants or trabeculectomy with MMC, performed as a primary procedure in 125 and 117 patients with OAG, respectively. Postoperative complications developed in 36 patients (29%) in the tube group and 48 (41%) in the trabeculectomy group (*P* = 0.06). They included serious complications requiring reoperation or producing a loss of 2 Snellen lines or more in 1 patient (1%) and 8 patients (7%), respectively (*P* = 0.03).

A broad range of serious complications have also been described following XEN implantation [31–34]. Ibanez-Munoz et al. [31] reported a 5.5% rate of implant extrusion, leading in one case to endophthalmitis. The lack of a stent in the MIMS procedure constitutes one of its greatest advantages because the absence of a foreign body may avoid such complications. Also, the presence of a stent in the anterior chamber poses a risk of corneal decompensation, as it may generate corneal endothelial cell loss. XEN stent implantation has been associated with hypotony and its vision-threatening sequelae in 8.7–24.6% of cases, with a choroidal detachment rate as high as 9.5% [19, 21, 35]. In our study, hypotony resulted in one case each of choroidal effusion and ciliary body detachment (0.8%). Both resolved after administration of topical atropine drops. The reported rate of glaucoma reoperation after XEN stent implantation varies. A review published by Buffault et al. [36] reported a 5.7% rate of repeated filtering surgery or cyclo-destructive procedure, and Heidinger et al. [37] reported a 14.1% rate of additional glaucoma surgery. Busch et al. [21] found that 43% of eyes treated with the XEN implant required needling. In our study, only 2 patients (2.1% of the cohort) required trabeculectomy following MIMS, and 1 required needling; both surgeries were deemed successful.

Intraoperative bleeding in glaucoma surgeries can promote inflammation or pressure spikes, leading to surgical failure. In addition, intraoperative IOP fluctuations can lead to intraocular bleeding (i.e., suprachoroidal hemorrhage), potentially leading to permanently poor outcomes and/or need for reoperation [38–40]. Trabeculectomy sclerostomy, tube lumens, as well as the channel created in MIMS procedure, may be obstructed by frank blood or a fibrous membrane from resolving heme, leading to reoperation and/or surgical failure. Although the evidence suggests antithrombotic agents can be continued in the setting of cataract surgery, there is no consensus on the management of these therapies in glaucoma surgery. The decision to exclude patients who are unable to discontinue the use of anti-thrombotic agents in the setting of the study, was made in order to achieve standardization. However, in the absence of consensus, the authors think that in the future, the decision to alter medication regimens should be left for the surgeon discretion. It should be personalized to each patient’s indication for antithrombotic therapy [41].

The MIMS procedure has a short learning curve and is exceptionally quick to perform, with a mean duration of 2 min per procedure. As health expenditures for glaucoma treatment are spiraling [42, 43], and as every minute in the operating theatre is costly, the short procedure time, averaging at just 2:01 min, could potentially save valuable time in surgery, making MIMS a relatively cost-effective procedure in this aspect, especially when combined with cataract surgery.

When trying to figure out which glaucoma patient might benefit the most from MIMS operation, one needs to consider the final average IOP which was around 17.5 mmHg, which may not suffice for advanced glaucoma cases.

### Study limitations

The outbreak of COVID-19 and subsequent lockdowns caused patients to miss a relatively high percentage of study visits [44, 45]. An investigation of ophthalmic practices in 39 institutional centers in Italy by dell’Omo et al. [46] yielded 20,886 patients who underwent ocular surgery during lockdown compared to 55,259 and 56,640 in intra-and inter-year control periods, respectively. Only 70% of patients for whom an operation or intravitreal injection was recommended were actually operated; the remainder failed to attend because of fear of infection during hospitalization (23%), fear of taking public transportation (6.5%), or unavailability of swabs (0.5%). Elective surgeries were reduced by 96.2% and 96.4%, urgent surgeries by 49.7% and 50.2%, and intravitreal injections by 48.5% and 48.6% compared to intra-year and inter-year control periods, respectively. These findings, supported by the study of Yen et al. [47] (46.9% decrease in surgeries), could explain the 20.8% of our patients who did not adhere to the full follow-up protocol and were therefore dropped from the final data analysis (although they were included in the safety analysis). The investigators confirmed that there was a similar number of patients that were lost to follow-up following other surgical procedures performed during the study period. This, in turn, may have led to a negative bias in both efficacy and safety, as generally, patients with worse outcomes have a higher motivation to attend follow-up examinations.

Other limitations of our study were the absence of a comparator and the open-label design. Our results were compared to the literature, but the baseline characteristics of the patients with an indication for surgery in previously reported trials were highly variable in terms of glaucoma severity, IOP values (e.g., medicated and unmedicated), and number of glaucoma medications. Washout IOP values varied across studies, and not all studies, including ours, considered washout, mainly for ethical reasons. Randomized standardized controlled studies comparing MIMS to other filtration procedures are needed to further evaluate its efficacy and safety. The combined surgeries may also be another confounding factor, as phacoemulsification alone is known to reduce IOP to some extent. Lastly, a follow-up time of one year may be too short to evaluate sustained success. Therefore, longer-term studies are warranted.

## Conclusion

In conclusion, our 1-year results suggest that the MIMS procedure is relatively efficacious and safe for patients with OAG and uncontrolled IOP and may be considered in cases requiring surgical intervention.

## Data Availability

The datasets used and analysed during the current study are available from the corresponding author on reasonable request.
